# A GH89 human α-*N*-acetylglucosaminidase (hNAGLU) homologue from gut microbe *Bacteroides thetaiotaomicron* capable of hydrolyzing heparosan oligosaccharides

**DOI:** 10.1186/s13568-021-01253-1

**Published:** 2021-06-24

**Authors:** Xiaohong Yang, Xiaoxiao Yang, Hai Yu, Lan Na, Tamashree Ghosh, John B. McArthur, Tsui-Fen Chou, Patricia Dickson, Xi Chen

**Affiliations:** 1 Department of Chemistry, University of California, One Shields Avenue, Davis, CA 95616 USA; 2 Division of Biology and Biological Engineering, California Institute of Technology, Pasadena, CA 91125 USA; 3 Division of Genetics and Genomic Medicine, Department of Pediatrics, Washington University School of Medicine, St. Louis, MO 63110 USA; 4 Present Address: Rosalind Franklin Institute and University of Oxford, Harwell Campus, Didcot, OX11 0FA UK; 5 Present Address: Integrated Micro-Chromatography Systems, Inc, Irmo, SC 20963 USA

**Keywords:** α-*N*-Acetylglucosaminidase, NAGLU, Bacterial glycoside hydrolases, Heparosan oligosaccharides, *Bacteroides thetaiotaomicron*

## Abstract

Carbohydrate-Active enZYme (CAZY) GH89 family enzymes catalyze the cleavage of terminal α-*N*-acetylglucosamine from glycans and glycoconjugates. Although structurally and mechanistically similar to the human lysosomal α-*N*-acetylglucosaminidase (hNAGLU) in GH89 which is involved in the degradation of heparan sulfate in the lysosome, the reported bacterial GH89 enzymes characterized so far have no or low activity toward α-*N*-acetylglucosamine-terminated heparosan oligosaccharides, the preferred substrates of hNAGLU. We cloned and expressed several soluble and active recombinant bacterial GH89 enzymes in *Escherichia coli*. Among these enzymes, a truncated recombinant α-*N*-acetylglucosaminidase from gut symbiotic bacterium *Bacteroides thetaiotaomicron* ∆22Bt3590 was found to catalyze the cleavage of the terminal α1–4-linked *N*-acetylglucosamine (GlcNAc) from a heparosan disaccharide with high efficiency. Heparosan oligosaccharides with lengths up to decasaccharide were also suitable substrates. This bacterial α-*N*-acetylglucosaminidase could be a useful catalyst for heparan sulfate analysis.

## Key points

- Active GH89 recombinant bacterial homologues of human lysosomal α-*N*-acetylglucosaminidase (hNAGLU) are obtained.
- N-terminal truncation improves the soluble expression of several bacterial α-*N*-acetylglucosaminidases in *E. coli*.
- ∆22Bt3590 is expressed in *E. coli* at a level of 136 mg/L and is biochemically characterized.
- ∆22Bt3590 can catalyze the hydrolysis of heparosan oligosaccharides of different lengths.

## Introduction

α-*N*-Acetylglucosaminidases (EC 3.2.1.50) are glycoside hydrolases (GH) that catalyze the cleavage of the terminal *N*-acetylglucosamine from α-linked *N*-acetylglucosaminides (GlcNAcαOR). They have been grouped in the Carbohydrate-Active enZYme (CAZY) database (www.cazy.org) (Henrissat 1991) GH89 family based on their protein sequence similarity. Among more than 1000 predicted GH89 family members (>  100 from eukaryote and  >  900 from bacteria), only human α-*N*-acetylglucosaminidase (hNAGLU) (Weber et al. 1996) and its homologues from bacteria *Clostridium perfringens* ATCC 13124 (Ficko-Blean et al. 2008), *Clostridium perfringens* strain 13 (Fujita et al. 2011), and *Bifidobacterium bifidum* JCM1254 (Shimada et al. 2015) have been characterized.

hNAGLU is a lysosomal enzyme that catalyzes the hydrolysis of the terminal α1–4-linked *N*-acetylglucosamine (GlcNAc) at the non-reducing end of heparan sulfate (HS) (Birrane et al. 2019; Valstar et al. 2010). HS molecules are long unbranched negatively charged glycosaminoglycan (GAG) polysaccharides with disaccharide repeating units comprising an amino sugar and a uronic acid (Cartmell et al. 2017; Wang et al. 2010). Its biosynthesis in eukaryotes involves the formation of heparosan, a linear polysaccharide of a disaccharide repeating unit of –4GlcNAcα1–4GlcAβ1–, by extending from a tetrasaccharide core on proteoglycans followed by post-glycosylational modifications (Yu and Chen 2007) including GlcNAc *N*-deacetylation and *N*-sulfation, GlcA C5-epimeration, GlcA/IdoA 2–*O*–sulfation, glucosamine 6–*O*–sulfation and 3–*O*–sulfation (Esko and Lindahl 2001). Deficiency of hNAGLU causes a lysosomal storage disorder (LSD) (Platt 2018) called mucopolysaccharidosis type IIIB (MPS IIIB) or Sanfilippo syndrome B (Sanfilippo type B; MIM 252920) (Yogalingam and Hopwood 2001; Yogalingam et al. 2000). More than 150 MPS IIIB-causing mutations in the human *NAGLU* gene have been identified (Andrade et al. 2015). The crystal structure of the apo form of a recombinant human NAGLU (rhNAGLU, PDB ID: 4XWH) expressing high-mannose type *N*-glycans was reported recently (Birrane et al. 2019).

On the other hand, hNAGLU homologue from *Clostridium perfringens* ATCC 13124 (CpGH89) shares 28.2% identity with hNAGLU. Its crystal structures with or without β-GlcNAc and the crystal structure of its E483Q and E601Q double mutant in complex with GlcNAcα1–4Gal disaccharide have also been determined (PDB IDs: 2VCC, 2VCA, and 4A4A) (Ficko-Blean and Boraston 2012; Ficko-Blean et al. 2008). However, although also an α-*N*-acetylglucosaminidase, CpGH89 was reported to recognize terminal GlcNAcα1–4Gal motif in synthetic oligosaccharides and class III mucin glycans (Fujita et al. 2011), which is different from the GlcNAcα1–4GlcA and/or GlcNAcα1–4IdoA component in the substrates that is recognized by hNAGLU (Ficko-Blean and Boraston 2012). A similarly substrate specificity of the α-*N*-acetylglucosaminidase from *Bifidobacterium bifidum* JCM1254 (BfAgnB) in recognizing GlcNAcα1–4Gal-containing oligosaccharides and class III mucin glycans was also reported (Shimada et al. 2015).

To look for a bacterial homologue of hNAGLU which can catalyze the cleavage of the terminal α-linked GlcNAc in heparosan oligosaccharides efficiently, in addition to testing the activity of CpGH89 and its loop-truncated mutant, we cloned and examined the activities of three other GH89 enzymes including Bf0576 from *Bacteroides fragilis* as well as Bt0438 and Bt3590 from *Bacteroides thetaiotaomicron*. Among these, Bt3590 was shown to be a highly active α-*N*-acetylglucosaminidase that can catalyze the hydrolysis of terminal GlcNAc from the non-reducing end of heparosan oligosaccharides with varied lengths. It is a promising candidate that can be used for chemoenzymatic sequencing of heparin/HS oligosaccharides (Merry et al. 1999; Turnbull 2001; Turnbull et al. 1999).

## Materials and methods

### Bacterial strains, plasmids, and materials

*Escherichia coli* DH5α chemically competent cells were from Invitrogen (Carlsbad, CA). Genomic DNAs of *Bacteroides fragilis* NCTC 9343 (ATCC#25285), *Bacteroides thetaiotaomicron* VPI-5482 (ATCC#2914D-5), and *Clostridium perfringens* (ATCC#13124) were from American Type Culture Collection (ATCC, Manassas, VA, USA). Expression vector pET15b was from Novagen (EMD Biosciences Inc., Madison, WI, USA). Bio-Scale Mini Nuvia IMAC Cartridge and Bio-Scale™ Mini Bio-Gel^®^ P-6 Desalting Cartridge were from Bio-Rad (Hercules, CA, USA). AccuPrep^®^ PCR/Gel purification kit was from BIONEER Corporation. GeneJET plasmid spin kit, 1 kb DNA ladder, pre-stained protein ladder and FastDigest *BamH*I and *Xho*I restriction enzymes were from Fisher Scientific (Tustin, CA, USA). Phusion^®^ HF DNA polymerase, Q5^®^ site-directed mutagenesis kit, and T4 DNA ligase were from New England Biolabs Inc. (Beverly, MA, USA). 4-Methylumbelliferyl 2-acetamido-2-deoxy-α-D-glucopyranoside (GlcNAcαMU, **1**) was from Toronto Research Chemicals (North York, Canada) and α-GlcNAc-terminated heparosan oligosaccharides **2–6** were synthesized previously using an efficient chemoenzymatic method (Na et al. 2020).

### Cloning of full-length and truncated α-*N*-acetylglucosaminidases from *B. fragilis*, *B. thetaiotaomicron*, and *C. perfringens*

The genes encoding the full-length Bf0576 from *B. fragilis*, Bt0438 and Bt3590 from *B. thetaiotaomicron*, and CpGH89 from *C. perfringens* were amplified by polymerase chain reactions (PCRs) from the corresponding genomic DNAs. Genes for truncated proteins ∆17Bf0576 (residues 18–718), ∆24Bt0438 (residues 25–730), and ∆22Bt3590 (residues 23–732) were amplified from the corresponding plasmids containing the full-length genes (see below for cloning) by PCRs. DNA sequence encoding loop (residues 680–686)-truncated CpGH89 (tCpGH89) was amplified from the plasmid containing the full-length CpGH89 (see below for cloning) using Q5 kit. The corresponding primers used are listed in Table 1. PCRs were performed in a 50 μL reaction mixture containing 20 ng of genomic DNA or 4 ng of plasmid as the template DNA, 1 μM each of forward and reverse primers, 5 μL of 10 × Phusion^®^ HF buffer, 1 mM dNTP mixture, and 5 units (1 μL) of Phusion^®^ HF DNA polymerase. The reaction mixtures were subjected to 35 cycles of amplifications with an annealing temperature of 55 °C for Bf0576, Bt0438 and Bt3590, 62 °C for CpGH89, 65 °C for tCpGH89. For cloning the full-length genes and the genes encoding the *N*-terminal truncated recombinant proteins, the resulting PCR products were digested with the corresponding restriction enzymes introduced in the primers, purified, and ligated with predigested pET15b vector. For cloning tCpGH89, the resulting PCR products were purified, and ligated with KLD Enzyme Mix included in the Q5^®^ Site-Directed Mutagenesis Kit. The ligated product was transformed into chemically competent *E. coli *DH5α cells. Positive plasmids were sequenced and subsequently transformed into homemade BL21 (DE3) chemically competent cells. Selected clones were grown for protein expression.

**Table 1:** Primers used for cloning full-length and truncated bacterial α-*N*-acetylglucosaminidases

Primers	Oligonucleotides^a^	
CpGH89	Forward	5′TTGGCT*CTCGAG*GGTGTTGAAATTACGGAAGGGGTTACTGTAACTGC3′
	Reverse	5′AGCCAA*GGATCC*TTATGATTCATTTTCACCTAATATTTTATCCATATTAGTTACTGAATAACTTTCCATGGC3′
tCpGH89	Forward	5′CATTCAAAAATAGTTTATGATAAGAGTGAATTTGAA AAAGCTATTGAAATATTTGC3′
	Reverse	5′TATTCCAAAGCCTGGTCTTGCATTTATAATAGACTCAGC3′
Bf0576	Forward	5′GTGTGTCTCGAGATGAATCGTAAATCAATACT3′
∆17Bf0576	Forward	5′GTGTGT*CTCGAG*GCAATGGCTTCTCCGGTAAC3′
Bf0576	Reverse	5′GTGTGT*GGATCC*TTATTCAACCGCTTGCATCA3′
Bt0438	Forward	5′GTGTGT*CTCGAG*ATGAACAGACAATACTTCTA3′
∆24Bt0438	Forward	5′GTGTGT*CTCGAG*AGTAACCCAGTATTAGAACA3′
Bt0438	Reverse	5′GTGTGT*GGATCC*TTAATAAAAATATTGCATATATT3′
Bt3590	Forward	5′GTGTGT*CTCGAG*ATGAATCATAAATACCTATA3′
∆22Bt3590	Forward	5′GTGTGT*CTCGAG*ACAGGCCCTCCTGTATTAAA3′
Bt3590	Reverse	5′GTGTGT*GGATCC*TTATTGTGCTTTGGTAAAGT3′

^a^Restriction sites are italicized and underlined

### Protein expression, purification, and quantification

The plasmid-bearing *E. coli* cells were cultured in 1 L Luria–Bertani (LB) rich medium (10 g L^−1^ tryptone, 5 g L^−1^ yeast extract, and 10 g L^−1^ NaCl) supplemented with 100 μg mL^−1^ ampicillin at 37 °C with shaking. Generally, overexpression of the target protein was achieved by inducing the *E. coli *culture with 0.1 mM of isopropyl-1-thio-β-D-galactopyranoside (IPTG) at OD_600 nm_  =  0.8–1.0 and incubating at 20 °C for 20 h with vigorous shaking at 250 rpm in a C25KC incubator shaker (New Brunswick Scientific, Edison, NJ). Cells were collected by centrifugation at 5000×*g*, 4 °C for 30 min. The cell precipitation was resuspended in Tris–HCl buffer (100 mM, pH 8.0), and then lysed by homogenizer. Cell debris was removed by centrifugation at 8000×*g* and 4 °C for 30 min, and the enzymes were purified from the supernatant by Bio-Scale Mini Nuvia IMAC Cartridge following the manufacturer’s instructions. Eluted fractions were pooled and loaded onto Bio-Scale™ Mini Bio-Gel^®^ P-6 Desalting Cartridge to remove imidazole and then redissolved in Na_2_HPO_4_–NaH_2_PO_4_ buffer (0.1 M, pH 6.5). The expression of the recombinant proteins was examined by SDS-polyacrylamide gel electrophoresis (SDS-PAGE) performed in 12% Tris-glycine gels, and the protein concentration was determined by NanoDrop Lite spectrophotometer from Fisher Scientific (Tustin, CA, USA).

### Enzyme assays of α-*N*-acetylglucosaminidases using GlcNAcαMU (1) as the substrate

Enzymatic assays (20 μL total reaction volume) were performed in duplicate in Na_2_HPO_4_–NaH_2_PO_4_ buffer (0.1 M, pH 6.5) containing GlcNAcαMU (**1**, 1 mM). An enzyme selected from Δ17Bf0576 (0.49 μM), Δ24Bt0438 (0.048 μM), Δ22Bt3590 (0.012 μM), CpGH89 (0.38 μM), or tCpGH89 (0.38 μM) was added and the reactions were allowed to proceed at 37 °C for 20 min or 20 h and stopped by adding 40 μL methanol. Samples were centrifuged and the supernatants were analyzed at 315 nm by an Agilent ultra-high performance liquid chromatography (UHPLC) system equipped with a membrane on-line degasser, a temperature control unit (set at 30 °C), and a diode array detector using EclipsePlusC18 RRHD column (2.1 × 50 mm I.D., 1.8 μm particle size; Agilent). Mobile phase A was 0.1% trifluoroacetic acid (TFA) in water, and mobile phase B was acetonitrile. The system was pre-equilibrated with a running mobile phase composed of mobile phase A and mobile phase B (95/5, v/v) at a flow rate of 0.25 mL/min. After injection of the sample, compound separation was carried out with two-phase gradient elution steps (starting at 95% A  +  5% B at 0 min to 50% A  +  50% B at 4 min, then back to 95% A  +  5% B at 5 min with the run stopped at 5.1 min).

### Enzyme assays of α-*N*-acetylglucosaminidases using GlcNAcα1–4GlcAβProNHFmoc (2) as the substrate

Enzymatic assays (20 μL total reaction volume) were performed in duplicate in Na_2_HPO_4_–NaH_2_PO_4_ buffer (0.1 M, pH 6.5) containing GlcNAcα1–4GlcAβProNHFmoc (**2**, 1 mM). An enzyme selected from Δ17Bf0576 (0.15 mM), Δ24Bt0438 (0.036 mM), Δ22Bt3590 (0.003 mM), CpGH89 (0.11 mM), or tCpGH89 (0.11 mM) was added and the reactions were allowed to proceed at 37 °C for 1 h or 20 h and stopped by adding 40 μL methanol. Samples were centrifuged and the supernatants were analyzed at 254 nm by a Shimadzu LC-2010A high-performance liquid chromatography (HPLC) system equipped with a membrane on-line degasser, a temperature control unit (set at 30 °C), and a diode array detector using XBridge^®^ BEH Amide column (4.6 × 250 mm I.D., 5 μm particle size, Waters) protected with a C18 guard column cartridge. Mobile phase A was 0.1% formic acid in water, and mobile phase B was acetonitrile. The system was pre-equilibrated with running mobile phase composed of mobile phase A and mobile phase B (20/80, v/v) at a flow rate of 0.8 mL/min. After injection of the sample, compound separation was carried out in a four-phase procedure with an isocratic condition of 20% A  +  80% B during 0–5 min, a gradient to 45% A  +  55% B during 5.0–5.5 min, a gradient back to 20% A  +  80% B during 5.5–6.0 min, followed by a 2 min-isocratic condition until the run was stopped at 8 min.

### pH profile of Δ22Bt3590

Enzymatic assays (20 μL total reaction volume) were performed in duplicate at 37 °C for 20 min in a buffer (100 mM) with a pH in the range of 3.0–10.0, disaccharide **2** (1 mM), and Δ22Bt3590 (0.51 μM). Buffers used were: citric acid-sodium citrate, pH 3.0–6.5; Na_2_HPO_4_-citric acid, pH 7.0–7.5; Tris–HCl, pH 8.0–8.5; and glycine–NaOH, pH 9.0–10.0. Reactions were stopped by adding 40 μL methanol. Samples were centrifuged, and then analyzed by HPLC as described above.

### Temperature profile assays for Δ22Bt3590

Enzymatic assays (20 μL total reaction volume) were performed in duplicate in citric acid-sodium citrate buffer (0.1 M, pH 5.0) containing disaccharide **2** (1 mM) and Δ22Bt3590 (0.51 μM) at different temperatures: 10, 15, 20, 25, 30, 35, 37, 40, 45, 50, 55, and 60 °C. Reactions were allowed to proceed for 15 min and stopped by adding 40 μL methanol. Samples were centrifuged, and then analyzed by HPLC as described above.

### Thermostability assays for Δ22Bt3590

Δ22Bt3590 dissolved in citric acid-sodium citrate buffer (0.1 M, pH 5.0) was incubated at 25, 30, and 37 °C for 1 h, 4 h, 8 h, and 24 h, respectively. After incubation, enzymatic assays (20 μL total reaction volume) were performed in duplicate at 37 °C in a mixture containing disaccharide **2** (1 mM) and incubated Δ22Bt3590 (0.35 μM). Reactions were allowed to proceed for 20 min and stopped by adding 40 μL methanol to each reaction mixture. Samples were centrifuged, and then analyzed by HPLC as described above.

### Effects of divalent metal cations, EDTA, and a reducing reagent DTT on the activity of Δ22Bt3590

Enzymatic assays were carried out in duplicate at 37 °C for 20 min in a total volume of 20 μL in citric acid-sodium citrate buffer (0.1 M, pH 5.0) containing disaccharide **2** (1 mM), Δ22Bt3590 (0.39 μM), and 10 mM of CaCl_2_, CuSO_4_, MgCl_2_, MnCl_2_, NiSO_4_, ZnCl_2_, ethylenediaminetetraacetic acid (EDTA), or dithiothreitol (DTT). Reactions without metal ions, EDTA, or DTT were used as controls. The reactions were quenched by adding 40 μL methanol. Samples were centrifuged, and then analyzed by HPLC as described above.

### Kinetic studies of Δ22Bt3590

To obtain apparent kinetic parameters with GlcNAcαMU (**1**) as the substrate, Δ22Bt3590 (containing 0.001 μM) was incubated with various concentrations (0.005, 0.0066, 0.008, 0.01, 0.0125, 0.02, 0.04, 0.1 and 0.2 mM) of GlcNAcαMU (**1**) in duplicate at 30 °C for 10 min (conversion was controlled to below 25%) in a total volume of 40 μL in citric acid-sodium citrate buffer (0.1 M, pH 5.0). The reactions were quenched by adding 40 μL methanol followed by incubation in an ice bath. Samples were centrifuged and analyzed by UHPLC as described above.

To obtain apparent kinetic parameters with GlcNAcα1–4GlcAβProNHFmoc (**2**) as the substrate, Δ22Bt3590 (0.086 μM) was incubated with various concentrations (0.05, 0.1, 0.2, 0.4, 0.8, 1.0, 3.0, 5.0, 8.0 and 10.0 mM) of GlcNAcα1–4GlcAβProNHFmoc (**2**) in duplicate at 30 °C for 10 min in a total volume of 20 μL in citric acid-sodium citrate buffer (0.1 M, pH 5.0). The reactions were quenched by adding 40 μL methanol. Samples were centrifuged and analyzed by HPLC as described above.

The apparent kinetic parameters were obtained by fitting the experimental data (the average values of duplicate assay results) into the Michaelis–Menten equation using Grafit 5.0.

### Substrate specificity studies of Δ22Bt3590

All reactions were carried out in duplicate at 30 °C or 37 °C in citric acid-sodium citrate buffer (0.1 M, pH 5.0) containing GlcNAcαMU (**1**) or one of the heparosan oligosaccharides GlcNAcα1–4GlcAβ1–(4GlcNAcα1–4GlcAβ1-)_n_ProNHFmoc (n  =  0–4) (**2**–**6**) (1 mM). Reactions at 37 °C used 33 μg mL^−1^ Δ22Bt3590 and aliquots of samples were taken at 20 min, 4 h, and 24 h and stopped by adding 40 μL methanol. Reactions at 30 °C used 29 μg mL^−1^ Δ22Bt3590 for 1 h reactions and 217 μg mL^−1^ Δ22Bt3590 for 24 h reactions. Reactions were stopped by adding 40 μL methanol, centrifuged, and the supernatants were subjected to UHPLC (for reactions using GlcNAcαMU **1** as the substrate) or HPLC (for reactions using a heparosan disaccharide **2** as the substrate) methods as described above. For samples using a heparosan oligosaccharide selected from **3–6** as the substrate, the UHPLC system is used with an AdvanceBioGlycan column (1.8 μm particle, 2.1 × 150 mm, Agilent Technologies, CA) and monitored at 254 nm. Mobile phase A was 0.1% trifluoroacetic acid (TFA) in water, and mobile phase B was acetonitrile. The system was pre-equilibrated with a running mobile phase composed of mobile phase A and mobile phase B (10/90, v/v) at a flow rate of 0.5 mL/min. After injection of the sample, compound separation was carried out in a three-phase procedure with a gradient starting from 10% A  +  90% B at 0 min to 30% A  +  70% B at 9 min followed by another gradient back to 10% A  +  90% B for the duration of 9–9.5 min, then an isocratic duration till the run was stopped at 12.5 min.

## Results

### Cloning and expression of bacterial CAZy GH89 α-*N*-acetylglucosaminidases

**Fig. 1:**
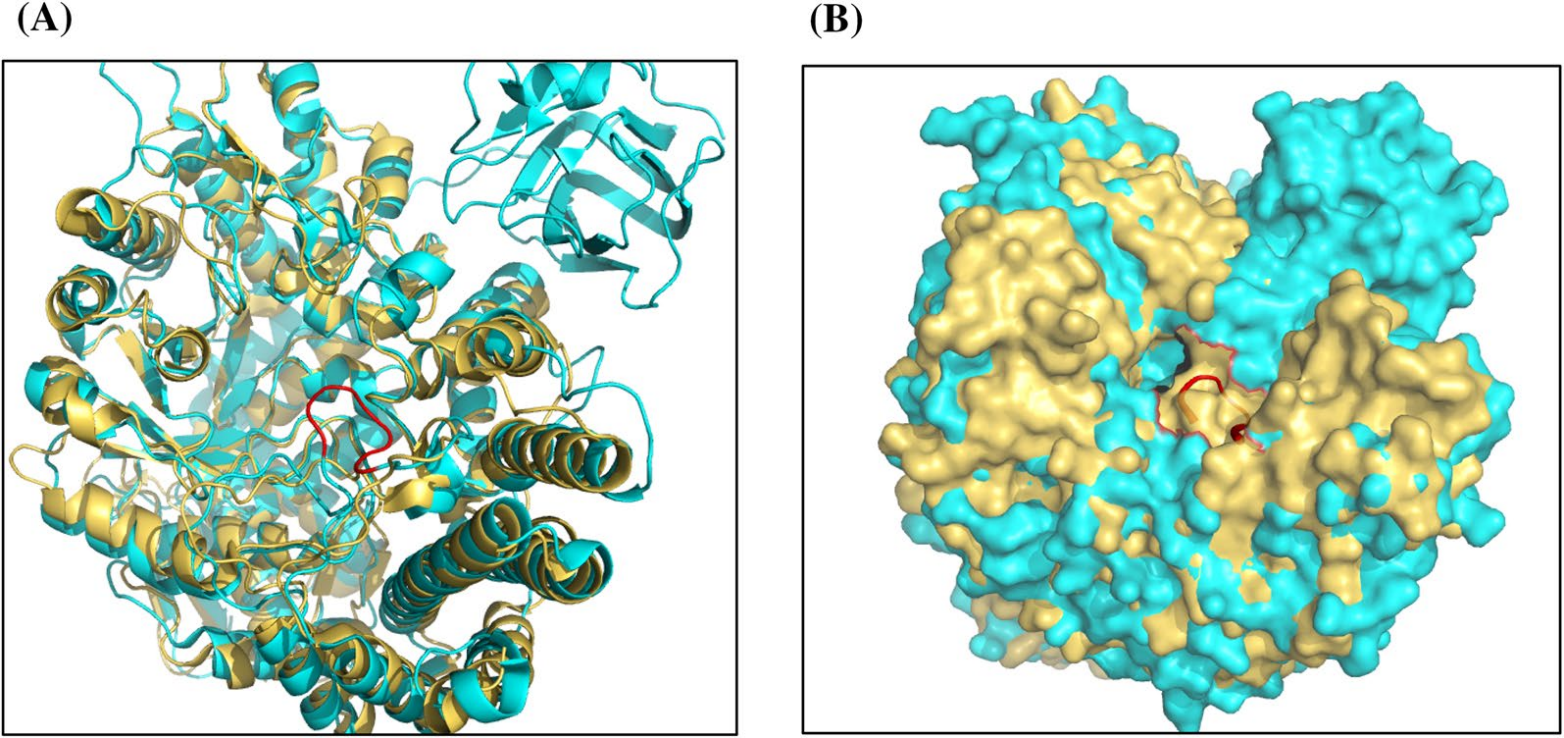
Structural alignment of hNAGLU (PDB ID: 4XWH, golden) and CpGH89 (PDB ID: 2VCC, cyan) in a cartoon (**A**) or a surface-filling (**B**) representation. An extra loop in CpGH89 (residues 680–686) containing W685, the key residue for the recognition of GlcNAcα1–4Gal motif in CpGH89 (Birrane et al. 2019; Ficko-Blean and Boraston 2012), was shown in red in a cartoon presentation. The figures were generated with PyMOL

Protein structure-based alignment using UCSF Chimera (Pettersen et al. 2004) and structural overlay using PyMOL (Yuan et al. 2016) of CpGH89 (GenBank accession number ABG84150.1) and hNAGLU reveal an extra loop in CpGH89 (residues 680–686) containing a tryptophan (W685) residue which was suggested to be important for the recognition of the GlcNAcα1–4Gal motif of its substrate (Fig. 1; Ficko-Blean and Boraston 2012). This loop was hypothesized to restrict the type of the substrate that can enter the binding pocket and cause the high substrate selectivity of CpGH89, preventing the binding of heparan sulfate-type substrate that containing a terminal GlcNAc α-linked to a β-D-glucuronic acid or α-L-iduronic acid (Birrane et al. 2019; Ficko-Blean and Boraston 2012). Therefore, a truncated CpGH89 (tCpGH89) with this extra loop deleted was designed and cloned.

**Fig. 2:**
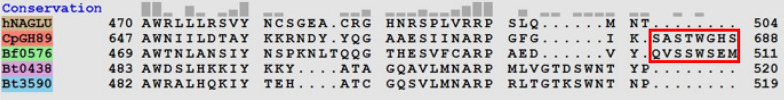
A segment of structure-based protein sequence alignment of α-*N*-acetylglucosaminidases including hNAGLU (GenBank accession no. AAB06188.1), CpGH89 (GenBank accession no. ABG84150.1), Bf0576 (GenBank accession no. CAH06355.1), Bt0438 (GenBank Accession No. AAO75545.1), and Bt3590 (GenBank Accession No. AAO78695.1). The Trp685-containing extra loop in CpGH89 structure and the corresponding predicted loop in Bf0576 structural model are shown in the red square

To identify potential bacterial hNAGLU homologues that can efficiently use HS as the substrate, protein sequence of hNAGLU (GenBank Accession Number AEE60931.1) was used to search for candidates in gut microbes that are known for their capability of using host HS as the major source of nutrients (Cartmell et al. 2017; Martens et al. 2008), Bf0576 from *B. fragilis* (GenBank Accession Number CAH06355.1) as well as Bt0438 (GenBank Accession Number AAO75545.1) and Bt3590 from *B. thetaiotaomicron* (GenBank Accession Number AAO78695.1) were identified. Protein sequence alignment of hNAGLU, CpGH89, Bf0576, Bt0438, and Bt3590 using the online server Clustal Omega (https://www.ebi.ac.uk/Tools/msa/clustalo/) showed that Bf0576, Bt0438, and Bt3590 share 34.6%, 32.8%, and 34.7% protein sequence identity with hNAGLU and 37.4%, 28.7%, and 30% sequence identity with CpGH89, respectively. The models of Bf0576, Bt0438, and Bt3590 generated by online server SWISS-MODEL (https://swissmodel.expasy.org/) were used for further structure-based sequence alignment with hNAGLU (PDB ID: 4XWH) and CpGH89 (PDB ID: 2VCC) using UCSF Chimera (Pettersen et al. 2004). The Trp-containing extra loop presented in CpGH89 could also be found in Bf0576 structural model but is absent from the structural models of Bt0438 and Bt3590 (Fig. 2).

**Fig. 3:**
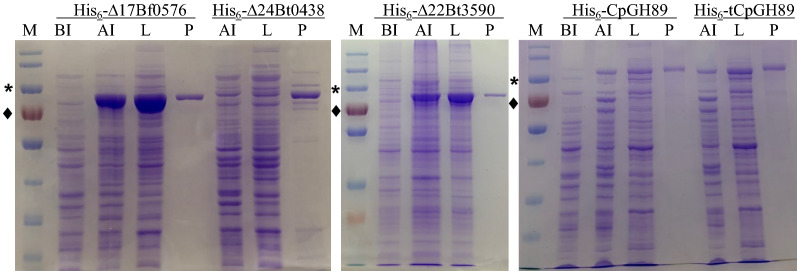
SDS-PAGE analysis of the expression and purification of recombinant bacterial α-*N*-acetylglucosaminidases including His_6_-CpGH89, and loop-deletion CpGH89 (His_6_-tCpGH89), His_6_-∆17Bf0576, His_6_-∆24Bt0438, His_6_-∆22Bt3590. Lanes: M, Thermo Scientific™ PageRuler™ Plus Prestained Protein Ladder (10–250 kDa) for His_6_-∆22Bt3590 samples and Thermo Scientific™ PageRuler™ Prestained Protein Ladder (10–180 kDa) for other samples. The size of the marker with an asterisk on the left is 100 kDa and the size of the marker with a diamond on the left is 75 kDa); BI, whole cell extract before induction; AI, whole cell extract after induction; L, lysate after induction; P, Ni^2+^-NTA column purified protein

Recombinant Bf0576, Bt0438, and Bt3590 were cloned into pET15b vector as N-His_6_-tagged proteins and expressed in BL21 (DE3). However, no significant expression of soluble proteins was observed (data not shown). After removing the potential transmembrane domain predicted by TMHMM Server v. 2.0 (http://www.cbs.dtu.dk/services/TMHMM/) at the N-terminus of each enzyme, ∆17Bf0576, ∆24Bt0438, and ∆22Bt3590 lacking the N-terminal 17, 24, and 22 amino acid residues were constructed and overexpressed. Soluble recombinant proteins were readily purified by nickel-nitrilotriacetic acid (Ni^2+^-NTA) affinity chromatography with yields of 170 mg, 9 mg, and 136 mg per liter culture with expected molecular weights of about 83 kDa, 86 kDa, and 86 kDa for ∆17Bf0576, ∆24Bt0438, and ∆22Bt3590 (Fig. 3), respectively. Full length CpGH89 and the loop (residues 680–686)-truncated tCpGH89 (expected molecular weights of 104.8 kDa and 104.1 kDa, respectively) (Fig. 3) were expressed under similar conditions with yields of 22 mg and 24 mg per liter, respectively.

### Activity assays of bacterial CAZy GH89 α-*N*-acetylglucosaminidases

**Fig. 4:**
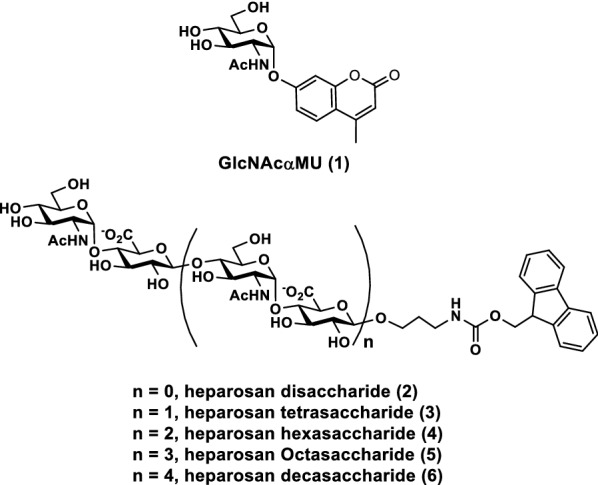
Structures of substrates (**1–6**) used for α-*N*-acetylglucosaminidase activity assays

The activities of recombinant bacterial α-*N*-acetylglucosaminidases were assayed using a commercially available fluorophore-tagged substrate, 4-methylumbelliferyl α-*N*-acetylglucosaminide (GlcNAcαMU, **1**) (Fig. 4), in a quantitative ultra-high performance liquid chromatography (UHPLC) assay with a diode array detector. As shown in Table 2, all recombinant α-*N*-acetylglucosaminidases tested were able to catalyze the cleavage of GlcNAcαMU at pH 6.5, with the highest efficiency observed for ∆22Bt3590, followed by ∆24Bt0438 with a medium relative catalytic efficiency. CpGH89, tCpGH89, and ∆17Bf0576 had similar relative catalytic efficiencies with 24.9–25.5% yields in 20 min when 0.38 μM (for CpGH89 or tCpGH89) or 0.49 μM (for ∆17Bf0576) of enzyme was used. In comparison, ∆24Bt0438 had a higher yield of 36.6 ± 1.8% in 20 min when it was used at an eight–ten-fold lower enzyme concentration (0.048 μM). ∆22Bt3590 had the highest efficiency with a yield of 38.7 ± 0.3% when 0.012 μM of enzyme (32–41-fold less) was used. All reactions went to completion when the reaction time was extended to 20 h.

**Table 2:** Activity comparison of recombinant bacterial α-*N*-acetylglucosaminidases in catalyzing the cleavage of GlcNAcαMU (**1**) at pH 6.5 and 37 °C

Enzyme	Concentration of catalyst (μM)	Conversion (%)	
		20 min	20 h
CpGH89	0.38	25.2 ± 0.4	100
tCpGH89	0.38	24.9 ± 0.4	
∆17Bf0576	0.49	25.5 ± 1.5	
∆24Bt0438	0.048	36.6 ± 1.8	
∆22Bt3590	0.012	38.7 ± 0.3	

Taking advantage of a previously synthesized fluorophore-labeled heparosan disaccharide GlcNAcα1-4GlcAβProNHFmoc (**2**) (Na et al. 2020), the activities of the recombinant enzymes in catalyzing the cleavage of the terminal α1–4-linked GlcNAc were assayed at pH 6.5. As shown in Table 3, although all enzymes were active and more than 91% of the substrate could be cleaved in 20 h, the concentrations of CpGH89 (0.11 mM), tCpGH89 (0.11 mM), and ∆17Bf0576 (0.15 mM) used were extremely high. When ∆24Bt0438 was used at 0.036 mM which was also a relatively high concentration, low yields of 12.2 ± 0.8% were achieved. In comparison, ∆22Bt3590 was able to catalyze the cleavage quite efficiently. When it was used at 0.003 mM, a concentration that was 12-fold lower than that of the ∆24Bt0438 and 37–50-fold lower than others, yields of 42.9 ± 1.2% were achieved, which were about 3.5-fold higher than that of the ∆24Bt0438. These results indicated that among the five recombinant enzymes, ∆22Bt3590 was the most efficient in catalyzing the cleavage of the terminal α1–4-linked GlcNAc from the heparosan disaccharide GlcNAcα1–4GlcAβProNHFmoc (**2**) at pH 6.5.

**Table 3:** Activity comparison of recombinant bacterial α-*N*-acetylglucosaminidases in catalyzing the cleavage of GlcNAcα1–4GlcAβProNHFmoc (**2**) at pH 6.5 and 37 °C

Catalyst	Concentration of catalyst (mM)	Conversion (%)	
		1 h	20 h
CpGH89	0.11	13.9 ± 0.7	96.0 ± 0.4
tCpGH89	0.11	23.7 ± 1.9	100
∆17Bf0576	0.15	66.9 ± 0.6	100
∆24Bt0438	0.036	12.2 ± 0.8	91.4 ± 0.6
∆22Bt3590	0.003	42.9 ± 1.2	97.0 ± 0.4

### pH profile of ∆22Bt3590 activity

**Fig. 5:**
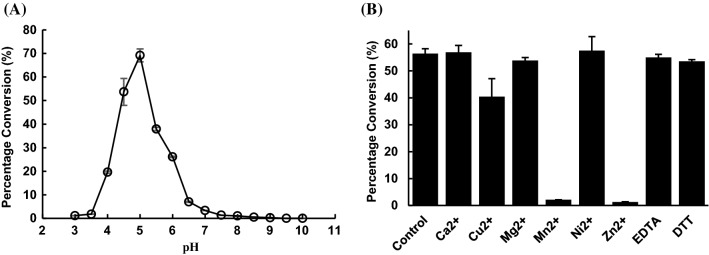
pH profile (**A**) and the effects of divalent metal ions, EDTA, and DTT on the activity of ∆22Bt3590 (**B**)

Using GlcNAcα1–4GlcAβProNHFmoc (**2**) as the substrate, ∆22Bt3590 was further characterized for its pH profile. It preferred an acidic pH and the optimal activity was at pH 5.0 in sodium citrate buffer (Fig. 5A). Its activity decreased dramatically when the pH fell below 4.0 or rose above 6.0.

### Effect of divalent metal cations, ethylenediaminetetraacetic acid (EDTA), and dithiothreitol (DTT) on the activity of ∆22Bt3590

The effects of various metal ions, the chelating reagent EDTA, and the reducing reagent DTT on the enzyme activity of ∆22Bt3590 were examined at pH 5.0. Reactions without metal ions were used as controls. As shown in Fig. 5B, a divalent metal cation was not required for the catalytic activity of ∆22Bt3590 as 10 mM of EDTA had no effect. Nevertheless, the presence of 10 mM CuCl_2_ decreased the reaction yields of ∆22Bt3590 slightly and the addition of MnCl_2_ or ZnCl_2_ almost abolished its activity completely. On the other hand, no significant effect in the activity of ∆22Bt3590 was observed for the reducing reagent DTT.

### Temperature profile studies of ∆22Bt3590

**Fig. 6:**
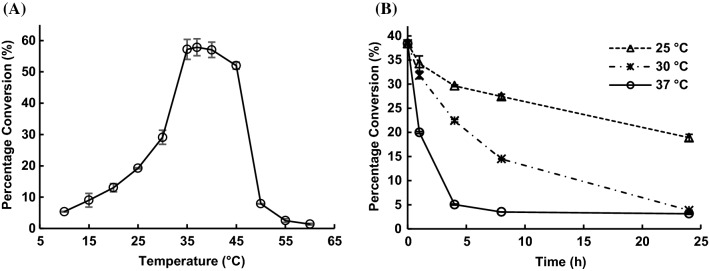
Temperature profiles (**A**) and thermostability (**B**) of ∆22Bt3590

∆22Bt3590 was shown to have optimal activities in the temperature range of 35–40 °C (Fig. 6A) and about 90% of the optimal activity was observed at 45 °C. Its activity decreased dramatically when the temperature reached 50 °C or higher. About 50% of the optimal activity was observed at 30 °C and the activity decreased with the decrease of the temperature. Minimal activity was observed at 10 °C.

### Thermostability studies of ∆22Bt3590

Thermostability studies of ∆22Bt3590 by incubating it at different temperatures (25 °C, 30 °C, and 37 °C) for different durations (1 h, 4 h, 8 h, and 24 h) in sodium citrate buffers (0.1 M, pH 5.0) showed (Fig. 6B) that the enzyme retained 89%, 83%, and 52% activities, respectively, after incubation at 25 °C, 30 °C, and 37 °C, for 1 h and 77%, 58%, and 13% activities, respectively, after incubation at 25 °C, 30 °C, and 37 °C, for 4 h. Incubating ∆22Bt3590 at 37 °C for 8 h or more almost abolished its activity while 71% and 38% activities retained, respectively, after incubation at 25 °C and 30 °C for 8 h. Incubation of the enzyme at 30 °C for 24 h also abolished its activity while 49% activity retained even after incubating the enzyme for 24 h at 25 °C.

### Apparent kinetic parameters of ∆22Bt3590

As shown in Table 4, the *K*_*M*_ value of ∆22Bt3590 for GlcNAcαMU (**1**) (4.6 ± 0.4 μM) obtained was much lower than those obtained for hNAGLU when GlcNAcαMU (0.17–0.33 mM) (Birrane et al. 2019; Zhao and Neufeld 2000), GlcNAcα*p*NP or two other aryl 2-acetamido-2-deoxy-α-glucosides and UDP-GlcNAc (0.14–0.74 mM) were used as the substrates (FIGURA 1977), and much less than that of CpGH89 using GlcNAcα*p*NP (1.1 mM) or 2,4-dinitrophenyl α-*N*-acetyl-D-glucosaminide (GlcNAcαDNP, 0.74 mM) as the substrates (Ficko-Blean et al. 2008). Compared to GlcNAcαMU (**1**), GlcNAcα1–4GlcAβProNHFmoc (**2**) was a less preferred substrate for ∆22Bt3590 which showed a much higher *K*_*M*_ value (2.19 ± 0.16 mM) than that for GlcNAcαMU (**1**) (*K*_M_ = 4.6 ± 0.4 μM), which led to about 490-fold lower *k*_cat_/*K*_M_ value (1.63 s^−1^ mM^−1^) when GlcNAcα1–4GlcAβProNHFmoc (**2**) was used as the substrate for ∆22Bt3590.

**Table 4:** Apparent kinetic parameters of ∆22Bt3590

Substrates	GlcNAcαMU (**1**)	GlcNAcα1–4GlcAβProNHFmoc (**2**)
*K*_M_ (mM)	(4.6 ± 0.4) × 10^−3^	2.19 ± 0.16
*k*_cat_ (s^−1^)	3.68 ± 0.07	3.57 ± 0.09
*k*_cat_/*K*_M_ (s^−1^ mM^−1^)	8.0 × 10^2^	1.63

### Substrate specificity studies of ∆22Bt3590

**Fig. 7:**
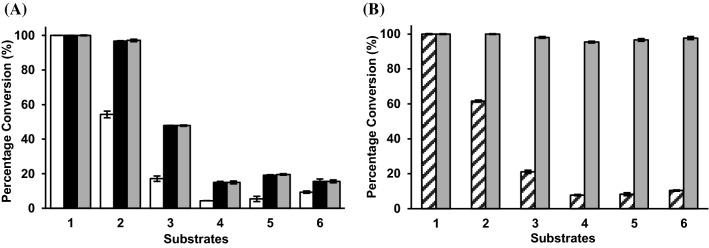
Substrate specificities of ∆22Bt3590 at 37 °C (**A**) or 30 °C (**B**) using GlcNAcαMU (**1**) and synthetic α-GlcNAc-terminated fluorescent-tagged heparosan oligosaccharides of varied lengths (**2–6**, Fig. 4) as potential substrates. Reaction times vary from 20 min (white columns), 1 h (patten filled columns), 4 h (black columns), to 24 h (gray columns)

Using GlcNAcαMU (**1**) and synthetic α-GlcNAc-terminated fluorophore-tagged heparosan oligosaccharides of varied lengths (**2–6**, Fig. 4) (Na et al. 2020) as substrates, substrate specificity studies of ∆22Bt3590 at 37 °C showed that heparosan oligosaccharides with longer lengths were poorer substrates than heparosan disaccharide **2** (Fig. 7A) and the yield of the catalytic reactions, in general, decreased with the increase of the substrate length. In agreement with the thermostability study results, ∆22Bt3590 lost most of its activity after 4 h-incubation at 37 °C as no further yield improvement was observed for the reactions with 24 h incubation compared to those with 4 h incubation time.

When the reaction temperature was decreased to 30 °C where ∆22Bt3590 was more stable (Fig. 6), an incubation time of 24 h was able to improve the reaction yields to reach more than 95% completion for all substrates tested (Fig. 7B).

## Discussion

*Bacteroides thetaiotaomicron* is a Gram-negative gut symbiotic bacterium which is well known for containing a large number of glycoside hydrolases and its capability of using different polysaccharides as nutrients (Cartmell et al. 2017; Xu and Gordon 2003). The complete 6.26-Mb genome sequence of *B. thetaiotaomicron* strain VPI-5482 (ATCC#29148) (Comstock and Coyne 2003; Xu et al. 2003) was predicted by PULDB database (http://www.cazy.org/PULDB/) (Terrapon et al. 2015, 2018) to encode more than 100 glycoside hydrolases responsible for breaking down a wide variety of polysaccharides. Nevertheless, among enzymes in *B. thetaiotaomicron* that are predicted to be responsible for glycosaminoglycan degradation (Ahn et al. 1998; Hooper et al. 2002; Ndeh et al. 2018, 2020), only polysaccharide lyases (PLs) and a GH88 ∆4,5-unsaturated uronyl hydrolase (Bt4658) have been biochemically characterized for using heparin and HS as high-priority nutrient sources by *B. thetaiotaomicron* (Cartmell et al. 2017; Dong et al. 2012; Han et al. 2009; Luo et al. 2007; Ulaganathan et al. 2017; Xu et al. 2003). Although *B. thetaiotaomicron* hNAGLU homologues in CAZy GH89 family were predicted to be α-*N*-acetylglucosaminidases that are involved in HS degradation based on deduced protein sequences from the *B. thetaiotaomicron* genomic sequence (Comstock and Coyne 2003; Martens et al. 2008), none have been characterized so far. Here we provide evidence that Bt0438 and Bt3590 from *B. thetaiotaomicron* VPI-5482 (ATCC#29148) as well as Bf0576 from of *Bacteroides fragilis* NCTC 9343 (ATCC#25285) are α-*N*-acetylglucosaminidases. While their full-length proteins did not expressed well in *E. coli* BL21(DE3) in a pET15b vector, N-terminal truncation led to the successful expression of the recombinant proteins ∆17Bf0576 (170 mg/L culture), ∆24Bt0438 (9 mg/L culture), and ∆22Bt3590 (136 mg/L culture) as soluble and active enzymes. Among these three, ∆22Bt3590 was the most efficient in catalyzing the cleavage of the terminal α-GlcNAc from commercially available GlcNAcαMU (**1**) at pH 6.5. ∆22Bt3590 was also shown to be able to use synthetic heparosan oligosaccharides (**2–6**) with an α-GlcNAc at the non-reducing end as the substrates.

**Fig. 8:**
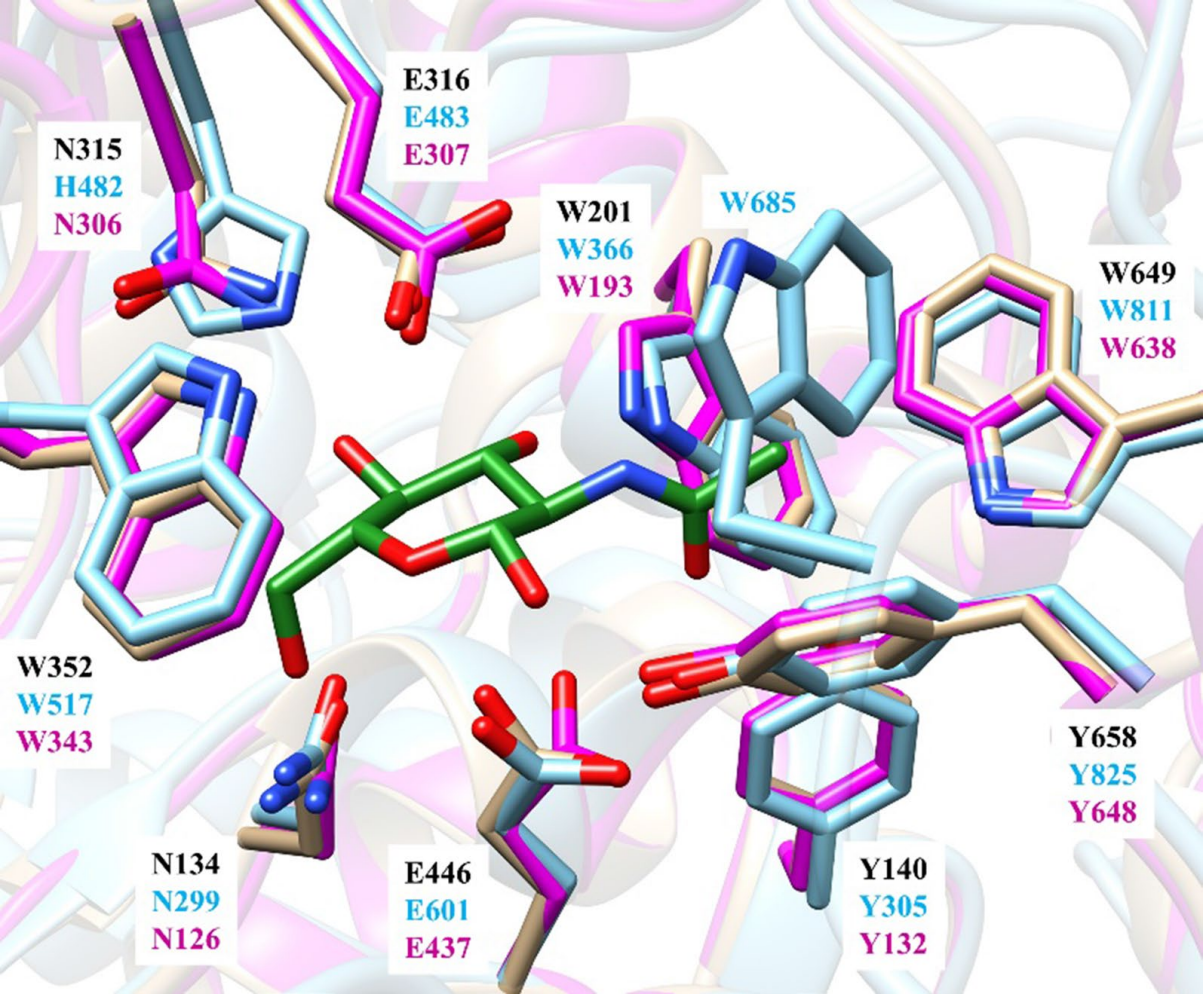
Structural overlay of the active sites of hNAGLU (PDB ID:4XWH, golden), CpGH89 (cyan) in complex with GlcNAc (green) (PDB ID: 2VCA), and Bt3590 model (magenta). The GlcNAc ligand (green) and the key residues in the active sites (labeled in black for hNAGLU, cyan for CpGH89, and magenta for Bt3590) are shown in stick representations. The figure was generated with UCSF Chimera (Pettersen et al. 2004)

A W638-containing loop in CpGH89 (Ficko-Blean et al. 2008; Yogalingam et al. 2000) that is absent in hNAGLU (Birrane et al. 2019) was suggested to be critical for the recognition of the specific GlcNAcα1–4GalβOR-type substrate by CpGH89. The presence of this loop introduces an extra tryptophan residue (Trp685) in the substrate-binding pocket of CpGH89 which is absent in hNAGLU (Fig. 8). Such a loop is also present in the structural model of Bf0576 but is absent from the structural models of Bt0438 and Bt3590. The loop-truncated version of CpGH89 (tCpGH89) showed a twofold higher activity in using GlcNAcα1–4GlcAβProNHFmoc (**2**) as the substrate compared to CpGH89.

While ∆22Bt3590 was the most reactive at 37 °C and pH 5.0 (Fig. 6A), it lost most of its activity after 4 h-incubation under this condition (Fig. 6B). In comparison, while at 30 °C ∆22Bt3590 was performing at 50% of its optimal activity (Fig. 6A), it was more stable and retained 38% activity even after 8 h-incubation under this condition (Fig. 6B). Indeed, ∆22Bt3590 was shown to be able to catalyze almost the complete cleavage of the terminal α-GlcNAc from heparosan disaccharide (**2**), tetrasaccharide (**3**), hexasaccharide (**4**), octasaccharide (**5**), and decasaccharide (**6**) at 30 °C within 24 h (Fig. 6B). In comparison, the cleavage of the terminal α-GlcNAc from heparosan oligosaccharides that was tetrasaccharide or larger (**3–6**) at 37 °C was incomplete even with up to 24 h-incubation time (Fig. 6A).

Unlike hNAGLU which has not been successfully expressed in *E. coli*, N-His_6_-tagged ∆22Bt3590 was readily expressed in *E. coli* as an active and soluble protein. About 136 mg protein was able to be purified from one liter of *E. coli* cell culture. Biochemical characterization of ∆22Bt3590 demonstrated that it had a similar optimal pH range (pH 4.5–5.0) and overall pH profile as hNAGLU (pH 4.2–4.8) (FIGURA 1977). ∆22Bt3590 could be a useful tool to replace hNAGLU in a strategy combining nitrous acid degradation and highly specific exolytic lysosomal enzymes for rapid and direct sequencing of heparin/HS saccharides (Merry et al. 1999; Turnbull 2001; Turnbull et al. 1999).

## Data Availability

All data generated or analyzed during this study are included in this published article.

## Abbreviations

CAZY: Carbohydrate-active enzyme
DTT: Dithiothreitol
EDTA: Ethylenediaminetetraacetic acid
GAG: Glycosaminoglycan
GlcNAc: *N*-Acetylglucosamine
GlcNAcαMU: 4-Methylumbelliferyl α-*N*-acetylglucosaminide
GlcNAcα*p*NP: *para*-Nitrophenyl α-*N*-acetylglucosaminide
hNAGLU: Human α-*N*-acetylglucosaminidase
HS: Heparan sulfate
IPTG: Isopropyl-1-thio-β-D-galactopyranoside
LB: Luria–Bertani
LSDs: Lysosomal storage disorders
MPS IIIB: Mucopolysaccharidosis type IIIB
NAGLU: α-*N*-Acetylglucosaminidase
Ni^2^^+^-NTA: Nickel-nitrilotriacetic acid
tCpGH89: Loop (residues 680–686)-truncated CpGH89
TFA: Trifluoroacetic acid
UHPLC: Ultra-high performance liquid chromatography

## References

[CR1] Ahn MY, Shin KH, Kim D-H, Jung E-A, Toida T, Linhardt RJ, Kim YS (1998). Characterization of a *Bacteroides* species from human intestine that degrades glycosaminoglycans. Can J Microbiol.

[CR2] Andrade F, Aldamiz-Echevarria L, Llarena M, Couce ML (2015). Sanfilippo syndrome: overall review. Pediatr Int.

[CR3] Birrane G, Dassier A-L, Romashko A, Lundberg D, Holmes K, Cottle T, Norton AW, Zhang B, Concino MF, Meiyappan M (2019). Structural characterization of the α-*N*-acetylglucosaminidase, a key enzyme in the pathogenesis of Sanfilippo syndrome B. J Struct Biol.

[CR4] Cartmell A, Lowe EC, Baslé A, Firbank SJ, Ndeh DA, Murray H, Terrapon N, Lombard V, Henrissat B, Turnbull JE, Czjzek M, Gilbert HJ, Bolam DN (2017). How members of the human gut microbiota overcome the sulfation problem posed by glycosaminoglycans. Proc Natl Acad Sci USA.

[CR5] Comstock LE, Coyne MJ (2003). *Bacteroides thetaiotaomicron*: a dynamic, niche-adapted human symbiont. BioEssays.

[CR6] Dong W, Lu W, McKeehan WL, Luo Y, Ye S (2012). Structural basis of heparan sulfate-specific degradation by heparinase III. Protein Cell.

[CR7] Esko JD, Lindahl U (2001). Molecular diversity of heparan sulfate. J Clin Invest.

[CR8] Ficko-Blean E, Boraston AB (2012). Structural analysis of a bacterial exo-alpha-D-*N*-acetylglucosaminidase in complex with an unusual disaccharide found in class III mucin. Glycobiology.

[CR9] Ficko-Blean E, Stubbs KA, Nemirovsky O, Vocadlo DJ, Boraston AB (2008). Structural and mechanistic insight into the basis of mucopolysaccharidosis IIIB. Proc Natl Acad Sci USA.

[CR10] Fujita M, Tsuchida A, Hirata A, Kobayashi N, Goto K, Osumi K, Hirose Y, Nakayama J, Yamanoi T, Ashida H, Mizuno M (2011). Glycoside hydrolase family 89 alpha-*N*-acetylglucosaminidase from *Clostridium perfringens* specifically acts on GlcNAc alpha1,4Gal beta1R at the non-reducing terminus of O-glycans in gastric mucin. J Biol Chem.

[CR11] Han YH, Garron ML, Kim HY, Kim WS, Zhang Z, Ryu KS, Shaya D, Xiao Z, Cheong C, Kim YS, Linhardt RJ, Jeon YH, Cygler M (2009). Structural snapshots of heparin depolymerization by heparin lyase I. J Biol Chem.

[CR12] Henrissat B (1991). A classification of glycosyl hydrolases based on amino acid sequence similarities. Biochem J.

[CR13] Hooper LV, Midtvedt T, Gordon JI (2002). How host-microbial interactions shape the nutrient environment of the mammalian intestine. Annu Rev Nutr.

[CR14] Luo Y, Huang X, McKeehan WL (2007). High yield, purity and activity of soluble recombinant *Bacteroides thetaiotaomicron* GST-heparinase I from *Escherichia coli*. Arch Biochem Biophys.

[CR15] Martens EC, Chiang HC, Gordon JI (2008). Mucosal glycan foraging enhances fitness and transmission of a saccharolytic human gut bacterial symbiont. Cell Host Microbe.

[CR16] Merry CL, Lyon M, Deakin JA, Hopwood JJ, Gallagher JT (1999). Highly sensitive sequencing of the sulfated domains of heparan sulfate. J Biol Chem.

[CR17] Na L, Yu H, McArthur JB, Ghosh T, Asbell T, Chen X (2020). Engineer *P. multocida* heparosan synthase 2 (PmHS2) for size-controlled synthesis of longer heparosan oligosaccharides. ACS Catal.

[CR18] Ndeh D, Munoz Munoz J, Cartmell A, Bulmer D, Wills C, Henrissat B, Gray J (2018). The human gut microbe *Bacteroides thetaiotaomicron* encodes the founding member of a novel glycosaminoglycan-degrading polysaccharide lyase family PL29. J Biol Chem.

[CR19] Ndeh D, Basle A, Strahl H, Yates EA, McClurgg UL, Henrissat B, Terrapon N, Cartmell A (2020). Metabolism of multiple glycosaminoglycans by *Bacteroides thetaiotaomicron* is orchestrated by a versatile core genetic locus. Nat Commun.

[CR20] Pettersen EF, Goddard TD, Huang CC, Couch GS, Greenblatt DM, Meng EC, Ferrin TE (2004). UCSF Chimera–a visualization system for exploratory research and analysis. J Comput Chem.

[CR21] Platt FM (2018). Emptying the stores: lysosomal diseases and therapeutic strategies. Nat Rev Drug Discov.

[CR22] Shimada Y, Watanabe Y, Wakinaka T, Funeno Y, Kubota M, Chaiwangsri T, Kurihara S, Yamamoto K, Katayama T, Ashida H (2015). alpha-*N*-Acetylglucosaminidase from *Bifidobacterium bifidum* specifically hydrolyzes alpha-linked *N*-acetylglucosamine at nonreducing terminus of O-glycan on gastric mucin. Appl Microbiol Biotechnol.

[CR23] Terrapon N, Lombard V, Gilbert HJ, Henrissat B (2015). Automatic prediction of polysaccharide utilization loci in *Bacteroidetes* species. Bioinformatics.

[CR24] Terrapon N, Lombard V, Drula E, Lapebie P, Al-Masaudi S, Gilbert HJ, Henrissat B (2018). PULDB: the expanded database of polysaccharide utilization loci. Nucleic Acids Res.

[CR25] Turnbull JE (2001). Integral glycan sequencing of heparan sulfate and heparin saccharides. Methods Mol Biol.

[CR26] Turnbull JE, Hopwood JJ, Gallagher JT (1999). A strategy for rapid sequencing of heparan sulfate and heparin saccharides. Proc Natl Acad Sci USA.

[CR27] Ulaganathan T, Shi R, Yao D, Gu RX, Garron ML, Cherney M, Tieleman DP, Sterner E, Li G, Li L, Linhardt RJ, Cygler M (2017). Conformational flexibility of PL12 family heparinases: structure and substrate specificity of heparinase III from *Bacteroides thetaiotaomicron* (BT4657). Glycobiology.

[CR28] Valstar MJ, Bruggenwirth HT, Olmer R, Wevers RA, Verheijen FW, Poorthuis BJ, Halley DJ, Wijburg FA (2010). Mucopolysaccharidosis type IIIB may predominantly present with an attenuated clinical phenotype. J Inherit Metab Dis.

[CR29] von FIGURA K (1977). Human α-*N*-Acetylglucosaminidase. Eur J Biochem.

[CR30] Wang Z, Ly M, Zhang F, Zhong W, Suen A, Hickey AM, Dordick JS, Linhardt RJ (2010). *E. coli* K5 fermentation and the preparation of heparosan, a bioengineered heparin precursor. Biotechnol Bioeng.

[CR31] Weber B, Blanch L, Clements PR, Scott HS, Hopwood JJ (1996). Cloning and expression of the gene involved in Sanfilippo B syndrome (mucopolysaccharidosis III B). Hum Mol Genet.

[CR32] Xu J, Gordon JI (2003). Honor thy symbionts. Proc Natl Acad Sci USA.

[CR33] Xu J, Bjursell MK, Himrod J, Deng S, Carmichael LK, Chiang HC, Hooper LV, Gordon JI (2003). A genomic view of the human-*Bacteroides thetaiotaomicron* symbiosis. Science.

[CR34] Yogalingam G, Hopwood JJ (2001). Molecular genetics of mucopolysaccharidosis type IIIA and IIIB: Diagnostic, clinical, and biological implications. Hum Mutat.

[CR35] Yogalingam G, Weber B, Meehan J, Rogers J, Hopwood JJ (2000). Mucopolysaccharidosis type IIIB: characterisation and expression of wild-type and mutant recombinant alpha-*N*-acetylglucosaminidase and relationship with sanfilippo phenotype in an attenuated patient. Biochim Biophys Acta.

[CR36] Yu H, Chen X (2007). Carbohydrate post-glycosylational modifications. Org Biomol Chem.

[CR37] Yuan S, Chan HCS, Filipek S, Vogel H (2016). PyMOL and inkscape bridge the data and the data visualization. Structure.

[CR38] Zhao K-W, Neufeld EF (2000). Purification and characterization of recombinant human α-*N*-acetylglucosaminidase secreted by Chinese hamster ovary cells. Protein Expr Purif.

